# Distal Pancreatic Resection for Neuroendocrine Tumors: Is Laparoscopic Really Better than Open?

**DOI:** 10.1007/s11605-015-2788-1

**Published:** 2015-03-11

**Authors:** Dimitrios Xourafas, Ali Tavakkoli, Thomas E. Clancy, Stanley W. Ashley

**Affiliations:** 1 Harvard School of Public Health, Boston, MA USA; 2 Brigham and Women’s Hospital, Harvard Medical School, Boston, MA USA

**Keywords:** Pancreatic neuroendocrine tumors, Distal pancreatectomy, Laparoscopic pancreatectomy

## Abstract

**Background:**

The latest studies on surgical and cost-analysis outcomes after laparoscopic distal pancreatectomy (LDP) highlight mixed and insufficient results. Whereas several investigators have compared surgical outcomes of LDP vs. open distal pancreatectomy (ODP) for adenocarcinomas, few similar studies have focused on pancreatic neuroendocrine tumors (PNETs).

**Methods:**

We reviewed the medical records of PNET patients undergoing distal pancreatectomy between 2004 and 2014. Patients were divided into LDP vs. ODP groups. Demographics, relevant comorbidities, oncologic variables, and cost-analysis data were assessed. Survival and Cox proportional hazards analyses were used to evaluate outcomes.

**Results:**

Of the 171 distal pancreatectomies for PNETs, 73 were laparoscopic, whereas 98 were open. Patients undergoing LDP demonstrated significantly lower rates of postoperative complications (*P* = 0.028) and had significantly shorter hospital stays (*P* = 0.008). On multivariable analysis, positive resection margins (*P* = 0.046), G3 grade (*P* = 0.036), advanced WHO classification (*P* = 0.016), TNM stage (*P* = 0.018), and readmission (*P* = 0.019) were significantly associated with poor survival; however, method of resection (LDP vs. ODP) was not (*P* = 0.254). The median total direct costs of LDP vs. ODP did not differ significantly.

**Conclusions:**

In response to the recent considerable controversy surrounding the costs and surgical outcomes of LDP vs. ODP, our results show that LDP for PNETs is cost-neutral and significantly reduces postoperative morbidity without compromising oncologic outcomes and survival.

## Introduction

Pancreatic neuroendocrine tumors (PNETs) are rare, representing approximately 2–4 % of all pancreatic tumors with an incidence of 2–3 cases per million people.^1^^,^ 2 In contrast to pancreatic adenocarcinoma, PNETs encompass a wider biological spectrum, which grants them a poorly defined natural history and often a challenging prognosis.^3^^,^ 4 Surgery is the standard curative modality for PNETs; however, surgical and oncologic outcomes vary significantly depending on whether the tumor is single or multiple, benign or malignant, functioning or nonfunctioning or whether resectable metastatic disease to the liver is present.^3^^–^ 5 Laparoscopic distal pancreatectomy (LDP) is well suited for complete radical resection of PNETs located in the pancreatic body or tail, at least for tumors that are relatively small and solitary.

Compared to open distal pancreatectomy (ODP), LDP performed in well-selected groups of pancreatic lesions has been confirmed to have superior results in terms of intraoperative blood loss, postoperative pain, time to recovery, and length of hospital stay (LHS).^6^^–^ 9 Despite these findings, latest reports on surgical outcomes of LDP vs. ODP highlight relevant inconsistencies between the two techniques. These inconsistencies involve operating time, rates of postoperative complications, pancreatic fistula, spleen preservation and conversion, costs, but more importantly, oncologic outcomes.^10^^,^ 11

Several investigators claim that the results of the surgical outcomes of LDP, as opposed to ODP, are neither clearly demonstrated nor generalizable due to underpowered existing comparative studies, introduction of important selection biases, and the nationwide underuse of laparoscopic pancreatectomy.^12^^–^ 14 Additionally, little data exist on long-term oncologic outcomes and mortality rates between LDP vs. ODP; therefore, studies with larger sample sizes and longer follow-ups are needed to clarify these issues.^10^^,^ 11 In an era of limited resources, concerns have been also raised about increasing costs for the laparoscopic approach, due to prolonged operating time and the relatively high cost of disposable surgical devices.^15^

Many reports on surgical outcomes after LDP vs. ODP have focused exclusively on well-selected subsets of pancreatic lesions to ensure homogeneous sampling, especially given that tumor factors may affect a patient’s suitability for laparoscopic resection.^16^^,^ 17 These comparative cohorts have predominately analyzed LDP vs. ODP outcomes for pancreatic adenocarcinoma. Few large series have assessed similar outcomes for PNETs due to their heterogeneity and complex patterns of clinical behavior, which cause significant variability in oncologic outcomes and survival.^4^^,^ 5 ^,^ 18 To clarify mixed findings and to address recent salient controversies regarding postoperative, oncologic, and cost-analysis outcomes after LDP vs. ODP, we focused on a subset of PNET patients who underwent distal pancreatectomy at our institution.

## Materials and Methods

The study was approved by our Institutional Review Board (IRB). International Classification of Disease (ICD)-9 codes for PNETs were identified from the Research Patient Data Registry (RPDR). A selected subset of patients with surgically resected, pathologically confirmed PNETs between July 2004 and 2014 were identified. After initial review of medical records, we excluded from the final analysis patients with multiple endocrine neoplasia type I or Von Hippel–Lindau disease, given their association with multiple other neoplasms that require more than pancreatic resection for cure.

Descriptive data were collected by review of patient’s medical records. Preoperative variables included age, gender, race, and relevant comorbidities, such as history of smoking, alcohol use, and diabetes mellitus. Patients with signs, symptoms, and biochemical evidence of pancreatic hormonal excess were considered to have functioning tumors, which were histopathologically confirmed after resection, whereas patients with no recognizable clinical syndrome and normal serum hormone levels were considered to have nonfunctioning tumors. Preoperative assessment of serum Chromogranin A levels were also recorded using a cutoff range of 84–87 U/L to obtain a high specificity for PNETs.^19^ Operations were grouped as LDP vs. ODP with or without spleen preservation or liver resection.

Detailed baseline information on PNETs included tumor diameter and location. Distant metastasis was defined as liver metastases when only the liver was involved and liver and extra-hepatic metastases when bone, lung, or brain metastases were additionally demonstrated. Pathological characteristics included tumor grade, lymphovascular invasion, regional lymph node status, and resection margin status, which were determined from final pathology reports. Tumors were classified according to the World Health Organization system (WHO)^20^ and staged according to the TNM scheme, which has been proposed by the European Neuroendocrine Tumor Society (ENETS).^21^

Postoperative complications were gathered from daily progress notes and discharge summaries. Postoperative pancreatic fistula (POPF) was assessed in 98 % of patients in this cohort and defined as abdominal drainage with an amylase level >3 times the upper limit of normal after postoperative day number 3, according to the International Study Group on Pancreatic Fistula (ISGPF) recommendations. POPFs were additionally graded based on the ISGPF criteria as follows: biochemical fistulas without clinical sequelae were graded as A. Those requiring any therapeutic intervention were graded as B, and fistulas with severe clinical sequelae were graded as C.^22^ Abscess was diagnosed when culture-positive purulent drainage from an intra-abdominal fluid collection was obtained percutaneously or operatively and/or when fluid collection with systemic or localized signs of infection was confirmed radiologically. Wound infection was defined as any wound that required opening or antibiotics beyond standard prophylaxis.

R0 resection was considered when the primary tumor was removed with negative margins. Patients with microscopically or grossly positive margins were classified as having had an R1 or R2 resection, respectively. Conversion was defined as the need for an abdominal incision to deal with any intraoperative complication and allow completion of the case. LHS was calculated from date of operation to date of hospital discharge. Readmission was defined as re-hospitalization within 30 days from discharge. Perioperative mortality was defined as death within 30 days from the operation, or within the original hospital admission. Survival was calculated from the date of operation through the date of last follow-up.

For cost-analysis purposes, standard tariffs set by our hospital independent coding and costing committee were adopted. The cost per patient visit was retrieved using recorded information by patient medical record number, and specific visit number using the institution’s financial cost accounting system. Total direct costs were defined as the median cost across cases in each group, and included all costs associated with the inpatient encounter, from admission to discharge for all hospital services provided. For both the LDP and ODP groups, cost analysis included the following:

- Operating room (OR) time in minutes, which was defined as the time from which the patient entered the operating room to the time at which he exited
- OR costs including team costs driven by the duration of the case, supplies, and recovery room costs
- Intra- and postoperative blood transfusion requirement costs
- Nursing, pharmacy, and laboratory testing costs
- Postoperative imaging costs including radiological re-intervention within the same hospitalization

Other costs included anesthesia, cardiac noninvasive testing, emergency department costs, gastrointestinal endoscopy, nutrition services, OR special charges, physical and occupational therapy, radiation therapy, and respiratory/pulmonary therapy costs. Indirect costs were excluded from our calculations.

The 11 laparoscopic cases, which were converted to open, were classified under the LDP category according to an intent-to-treat analysis framework. Continuous variables were compared using Student’s *t* test or Wilcoxon rank-sum test, whereas categorical variables were compared using Pearson’s chi-squared test or Fisher’s exact test as appropriate. Survival probability was estimated using the Kaplan–Meier method and compared using the log-rank test. Univariate and multivariable analyses were performed using Cox proportional hazards models. For cost analysis, we compared ratios of median costs for each cost category and reported the percentage of change in median cost. Continuous variables are reported as mean ± standard deviation or median and range. Categorical variables are presented as numbers (*n*) and percentages (%). A *P* value of less than 0.05 was considered statistically significant. Statistical analyses were conducted using the SAS statistical software program version 9.2 (SAS Institute, Cary, NC).

## Results

We compared 73 PNET patients who underwent LDP to 98 patients who underwent ODP.

### Demographics and Clinical Characteristics

Demographics and clinical characteristics are described in Table 1. The median age of the entire cohort was 61 years (range 20–95) with 89 male (52 %) and 153 Caucasian patients (89 %). There were no statistically significant differences with respect to age, gender, race, body mass index (BMI), and relevant comorbidities between the two groups. Overall, 37 patients (22 %) had functioning tumors, including 29 insulinomas, 5 gastrinomas, 2 glucagonomas, and 1 adrenocorticotropic hormone (ACTH)-secreting tumor, whereas 134 patients (78 %) had nonfunctioning tumors. A statistically higher proportion of patients with functioning tumors underwent ODP, whereas those with nonfunctioning tumors more often underwent LDP (*P* = 0.010).

**Table 1:** Demographics and clinical characteristics of PNET patients undergoing distal pancreatic resection and location and size of their tumors

	LDP (*n* = 73)	ODP (*n* = 98)	*P* value
Median age, *n* (range)	61 (20–95)	62 (34–92)	0.334
Male gender, *n* (%)	41 (56 %)	48 (49 %)	0.358
Race, *n* (%):			
Caucasian	64 (88 %)	89 (91 %)	0.616
African American	2 (3 %)	3 (3 %)	0.901
Hispanic	2 (3 %)	1 (1 %)	0.397
Asian	5 (6 %)	5 (5 %)	0.630
Median BMI, *n*	27.8	28.4	0.756
History of smoking, *n* (%)	21 (28 %)	31 (31 %)	0.687
Alcohol use, *n* (%)	20 (27 %)	22 (22 %)	0.457
Diabetes, *n* (%)	12 (16 %)	13 (13 %)	0.561
Tumor type, *n* (%):			
Nonfunctioning	64 (88 %)	70 (72 %)	0.010
Insulinoma	8 (11 %)	21 (21 %)	0.098
Gastrinoma	1 (1 %)	4 (4 %)	0.297
Glucagonoma	0	2 (2 %)	0.507
ACTHoma	0	1 (1 %)	0.386
Presence of signs or symptoms, *n* (%):			
Asymptomatic	52 (72 %)	50 (51 %)	0.007
Palpitations	0	1 (1 %)	0.386
Diarrhea	0	4 (4 %)	0.080
GI ulcers	1 (1 %)	0	0.426
Migratory erythema	0	1 (1 %)	0.386
Weight gain	0	1 (1 %)	0.386
Vague abdominal pain	9 (12 %)	18 (18 %)	0.284
Weight loss	3 (4 %)	1 (1 %)	0.186
Jaundice	1 (1 %)	0	0.426
Hypoglycemia	7 (10 %)	19 (20 %)	0.077
Hyperglycemia	0	1 (1 %)	0.386
Fatigue	0	2 (2 %)	0.507
High Chromogranin A levels, *n* (%)	28 (38 %)	34 (35 %)	0.622
Median lesion size, cm (range)	2.2 (0.2–13)	2.7 (0.4–15)	0.056
Location, *n* (%):			
Body	11 (15 %)	22 (22 %)	0.226
Tail	62 (85 %)	76 (78 %)	0.246

Overall, 69 patients (40 %) were symptomatic. A significantly lower percentage of symptomatic patients underwent LDP (28 vs. 49 %, LDP vs. ODP respectively, *P* = 0.007). Despite this, there were no statistically significant differences with regard to the type of symptoms between the two groups. Abdominal pain was most frequently present in patients undergoing LDP (12 %), whereas hypoglycemia was the most frequent symptom occurring in patients who had ODP (20 %). Sixty-two patients (36 %) had elevated serum Chromogranin A levels, but this was not significantly different between patients undergoing LDP vs. ODP (38 vs. 35 %, LDP vs. ODP, *P* = 0.622).

### Tumor Size and Location

The size and anatomic location of the resected PNETs are also described in Table 1. The median tumor diameter in patients undergoing LDP, as determined by pathology, was lower compared to the median tumor diameter of those who had ODP (2.2 vs. 2.7 cm, LDP vs. ODP, *P* = 0.056). Overall, 33 PNETs were located in the body of the pancreas (19 %), whereas 138 were located in the tail (81 %).

### Operations

Overall, 171 distal pancreatectomies were performed. Fifty-one patients undergoing LDP required a splenectomy compared to 60 patients who underwent ODPs (61 vs. 69 %, LDP vs. ODP, *P* = 0.260). Seven patients (4 %) had a simultaneous liver resection, of which two were performed during LDP, whereas five during ODP (3 vs. 5 %, LDP vs. ODP, *P* = 0.700). In 11 patients (15 %), a laparoscopic procedure was converted to laparotomy: six procedures were converted due to deep adhesions, which prevented visibility, and five others were converted due to intraoperative bleeding that could not be controlled laparoscopically. Although the OR time of LDPs was shorter compared to the OR time for ODPs, such difference was not statistically significant (352 vs. 409 min, LDP vs. ODP respectively, *P* = 0.065).

### Pathologic Characteristics

Fifty-four PNETs (74 %) of patients undergoing LDP were low-grade, 15 intermediate-grade (20 %), and 4 high-grade (6 %) vs. 71 low-grade (72 %), 20 intermediate-grade (21 %), and 7 high-grade (7 %) of those undergoing ODP (Table 2). Seventeen tumors resected with laparoscopic surgery (23 %) had microscopic evidence of lymphovascular invasion vs. 35 of those resected using the open technique (35 %). In the LDP group, 11 patients (15 %) had positive regional lymph nodes, and three patients (4 %) had distant metastases at the time of resection. In contrast, in the ODP group, 19 patients (19 %) had positive regional lymph nodes and 11 patients (11 %) had distant metastases. Five of the 14 patients with metastatic disease (36 %) underwent resection of their primary PNET only, whereas nine underwent synchronous liver resection or cryoablation (64 %). Overall, 162 patients (95 %) had complete resection (R0), whereas nine patients (5 %) had evidence of microscopic disease on the pancreatic margin (R1).

**Table 2:** Pathologic characteristics of the 171 resected PNETs

	LDP (*n* = 73)	ODP (*n* = 98)	*P* value
Grade, *n* (%):			
Low (G1)	54 (74 %)	71 (72 %)	0.824
Intermediate (G2)	15 (20 %)	20 (21 %)	0.982
High (G3)	4 (6 %)	7 (7 %)	0.760
Lymphovascular invasion, *n* (%)	17 (23 %)	35 (35 %)	0.094
Positive lymph nodes, *n* (%)	11 (15 %)	19 (19 %)	0.426
Distant metastasis, *n* (%)	3 (4 %)	11 (11 %)	0.093
Positive resection margins, *n* (%)	2 (3 %)	7 (7 %)	0.303
WHO classification, *n* (%):			
Well-differentiated tumor (WDT)	60 (82 %)	72 (74 %)	0.200
Well-differentiated carcinoma (WDCa)	9 (12 %)	19 (19 %)	0.296
Poorly differentiated carcinoma (PDCa)	4 (6 %)	7 (7 %)	0.760
TMN stage, *n* (%):			
Stage 1	34 (47 %)	38 (39 %)	0.348
Stage 2	28 (38 %)	37 (38 %)	0.100
Stage 3	8 (11 %)	15 (15 %)	0.499
Stage 4	3 (4 %)	8 (8 %)	0.356

According to the WHO classification, 132 patients (77 %) had WDT (82 vs. 74 %, LDP vs. ODP, *P* = 0.200), 28 patients (16 %) had WDCa (12 vs. 19 %, LDP vs. ODP, *P* = 0.296), and 11 patients (6 %) had PDCa (6 vs. 7 %, LDP vs. ODP, *P* = 0.760). In terms of TNM stage, overall 72 patients (42 %) had stage 1 disease, 65 patients (38 %) stage 2, 23 patients (13 %) stage 3, and 11 patients (7 %) stage 4 disease. Although the LDP group had more WDTs and lower stages of disease, the two groups did not differ significantly in terms of pathologic characteristics of their tumors (Table 2).

### Postoperative and Oncologic Outcomes

The overall surgical morbidity in terms of postoperative complications was 40 %. Sixteen patients (9 %) required reoperation due to: incisional hernia (*n* = 5), tumor recurrence (*n* = 7), necrotizing pancreatitis associated with intra-abdominal abscess (*n* = 2), small bowel obstruction (*n* = 1), and persistent pancreatic leak associated with hemorrhage (*n* = 1). Twenty-one patients (12 %) required readmission for the treatment of postoperative complications. Overall, there was one perioperative death (1.02 %) after ODP for WDCa.

**Fig. 1:**
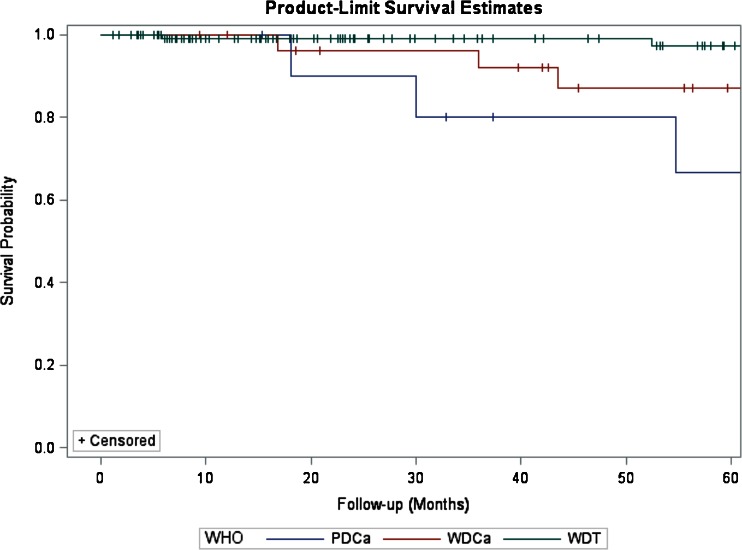
Kaplan–Meier estimates of survival for patients who underwent R0/R1 distal resection for PNETs. Five-year survival estimates stratified by WHO classification for patients with WDT, WDCa, and PDCa were 98.4, 71.4, and 36.3 %, respectively (log-rank test, *P* < 0.001)

**Fig. 2:**
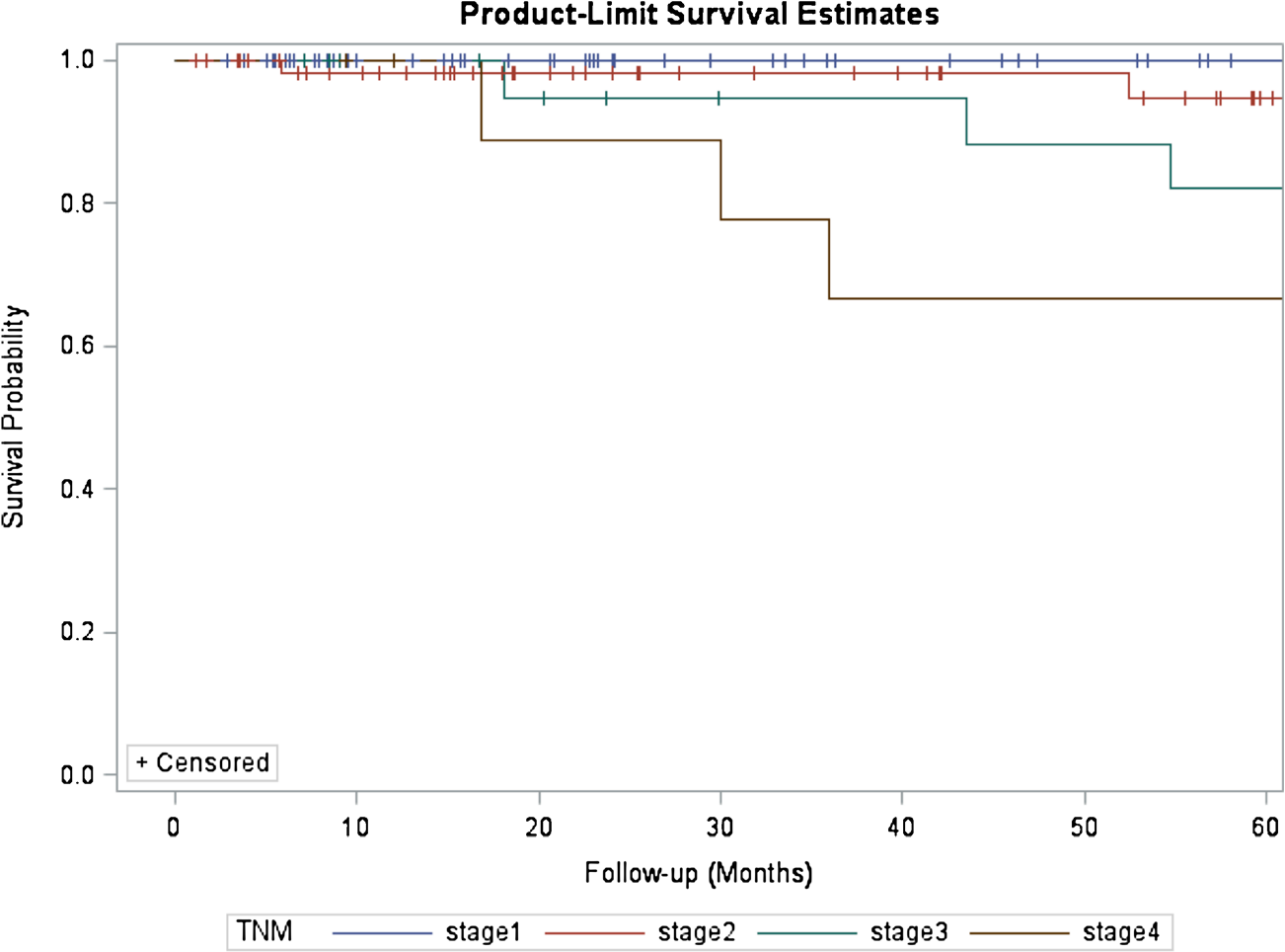
Five-year survival estimates stratified by ENETS-TNM stage for patients with stages 1, 2, 3, and 4 disease were 98.6, 95.3, 73.9, and 36.3 %, respectively (log-rank test, *P* < 0.001)

**Fig. 3:**
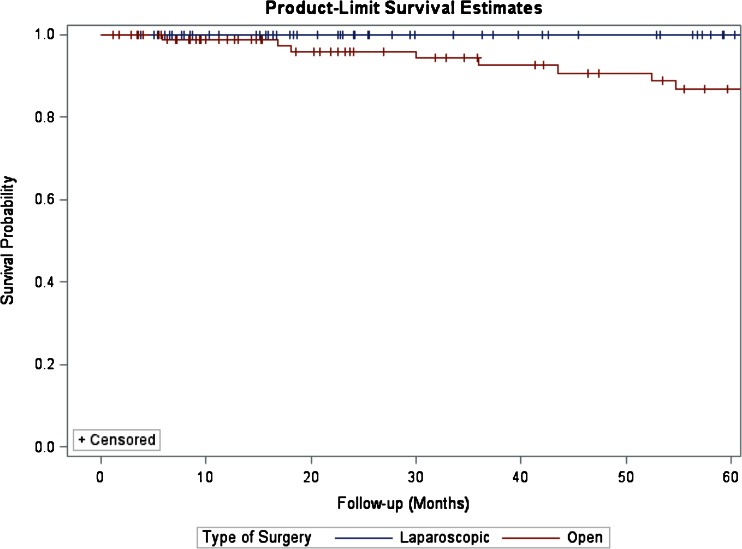
Kaplan–Meier estimates of survival for patients who underwent LDP vs. ODP for PNETs. Type of surgery (LDP vs. ODP) did not influence 5-year survival (log-rank test, *P* = 0.254)

For the entire cohort, the median LHS was 6 days (range of 3–39) and the median follow-up was 41 months (range of 2–254). There were no statistically significant differences in terms of follow-up between the two groups (32 vs. 44 months, LDP vs. ODP, *P* = 0.371). During this time, overall seven patients (4 %) developed recurrences. Five patients had a tumor recurrence in the liver, whereas two patients had recurrences in the liver and pancreatic bed. Recurrences occurred between a minimum of 17 and 108 months. The 5-year survival for the entire cohort was 95 %. The 5-year survival for patients with WDT, WDCa, and PDCa was 98.4, 71.4, and 36.3 %, respectively (*P* < 0.001, Fig. 1). The 5-year survival for patients with TNM stages 1, 2, 3, and 4 was 98.6, 95.3, 73.9, and 36.3 %, respectively (*P* < 0.001, Fig. 2). Type of surgery (LDP vs. ODP) did not influence the 5-year survival (log-rank test, *P* = 0.254, Fig. 3).

Although the most frequent major postoperative complication in both groups was POPF, we had no statistically significant differences in terms of frequency of occurrence (22 vs. 33 %, LDP vs. ODP, *P* = 0.168) or severity grade (grade A, 16 vs. 18 %, LDP vs. ODP, *P* = 0.840) between the two groups (Table 3). Despite having no significant differences with respect to individual type of complications (pancreatic fistula, incisional hernia, intra-abdominal collection, bowel obstruction, or superficial or deep would infection) between the LDP and ODP groups, we observed statistically significant differences in the overall rates of postoperative complications: fewer patients undergoing LDP developed postoperative complications (30 vs. 47 %, LDP vs. ODP, *P* = 0.028, Table 3).

**Table 3:** Postoperative outcomes for LDP vs. ODP for PNETs

	LDP (*n* = 73)	ODP (*n* = 98)	*P* value
Complications, *n* (%):			
Uncomplicated	51 (70 %)	52 (53 %)	0.028
Postoperative pancreatic fistula (POPF)	16 (22 %)	32 (33 %)	0.168
Incisional hernia	0	5 (5 %)	0.072
Intra-abdominal collection/abscess	6 (8 %)	5 (5 %)	0.531
Bowel obstruction	0	2 (2 %)	0.507
Wound infection (superficial or deep)	0	2 (2 %)	0.507
ISGPF pancreatic fistula, *n* (%):			
Grade A	12 (16 %)	18 (18 %)	0.840
Grade B	4 (6 %)	12 (13 %)	0.185
Grade C	0	2 (2 %)	0.507
Readmission, *n* (%)	9 (12 %)	19 (19 %)	0.296
Reoperation, *n* (%)	6 (8 %)	10 (10 %)	0.659
Overall mortality, *n* (%)	4 (5 %)	13 (13 %)	0.122
Median LHS, days (range)	5 (3–18)	7 (4–39)	0.008
Median follow-up, months (range)	32 (3–185)	44 (2–254)	0.371
Recurrence, *n* (%)	3 (4 %)	4 (4 %)	0.992

Additionally, for the entire cohort, we had no statistically significant differences between the two groups in terms of readmission rates (12 vs. 19 %, LDP vs. ODP, *P* = 0.296) or reoperation (8 vs. 10 %, LDP vs. ODP, *P* = 0.659). There were no perioperative deaths in the LDP group, but there was one death (1.02 %) in the ODP group (*P* = 0.386). Patients who underwent LDP had a significantly shorter median LHS than those undergoing ODP [5 days (range of 3–18) vs. 7 days (range of 4–39), LDP vs. ODP, *P* = 0.008]. With a median follow-up of 32 months (range of 3–185), three patients in the LDP group (4 %) developed a recurrence. Similarly, with a median follow-up of 44 months (range of 2–254), four patients in the ODP group (4 %) developed recurrences (*P* = 0.992). During follow-up, four patients (5 %) in the LDP group died vs. 13 patients (13 %) in the ODP group (*P* = 0.122), (Table 3).

### Potential Predictors of Poor Survival in Patients with PNETs

Additionally, we performed an analysis of potential prognostic factors impacting survival after distal pancreatic resection for PNETs. On univariate analysis (Table 4), tumor size >3 cm (*P* = 0.041) and positive resection margins (*P* = 0.016) predicted poor survival. Furthermore, tumor grade G-3 vs. G-1 (*P* = 0.041), WDCa (*P* = 0.003) and PDCa (*P* < 0.001) vs. WDT by WHO classification, and stages 3 (*P* = 0.014) and 4 (*P* < 0.001) vs. stage 1 by TMN, were also significantly associated with poor survival. In contrast, age >60 years (*P* = 0.231), male gender (*P* = 0.223), Caucasian race (*P* = 0.983), presence of symptoms (*P* = 0.937), functioning tumor type (*P* = 0.954), tumor location (either body or tail, *P* = 0.839), type of resection (LDP vs. ODP, *P* = 0.479), presence of postoperative complications (*P* = 0.390), readmission (*P* = 0.491), reoperation (*P* = 0.689), and recurrence (*P* = 0.996) did not correlate with poor survival.

**Table 4:** Univariate analysis of PNETs—clinicopathologic factors and survival

Variable	Hazard ratio (HR)	95 % CI for HR	*P* value
Age (>60)	0.556	0.213–1.454	0.231
Male	1.847	0.688–4.956	0.223
Caucasian	1.016	0.221–4.667	0.983
Presence of symptoms	0.961	0.357–2.586	0.937
Functioning tumor type	0.970	0.339–2.773	0.954
Tumor size >3 cm	4.681	1.065–20.572	0.041
Tumor location (tail)	2.805	0.366–2.477	0.839
Open surgery	1.513	0.480–4.771	0.479
High Chromogranin A levels	1.210	0.412–3.551	0.728
Positive resection margins	4.060	1.288–12.799	0.016
High-grade tumor (G3)	3.029	1.044–8.790	0.041
WDCa	10.041	2.093–48.173	0.003
PDCa	25.108	5.170–121.939	<0.001
TMN stage 3	14.642	1.693–126.591	0.014
TMN stage 4	63.680	7.286–556.531	<0.001
LHS >15 days	1.197	0.490–8.962	0.356
Presence of postoperative complications	1.523	0.583–3.978	0.390
Readmission	1.448	0.505–4.152	0.491
Reoperation	1.291	0.368–4.529	0.689
Presence of recurrence	1.004	0.130–7.760	0.996

On multivariable analysis when controlling for demographics and clinical characteristics—including age, gender, presence of symptoms, and functioning tumor type—positive resection margins (*P* = 0.046), tumor grade G-3 (*P* = 0.036), poorly differentiated tumors [WDCa (*P* = 0.002) and PDCa (*P* = 0.016)], TNM stage 3 (*P* = 0.032), TNM stage 4 (*P* = 0.018), and readmission (*P* = 0.019) remained significantly associated with poor survival (Table 5). On multivariable analysis, tumor >3 cm was no longer associated with poor survival (*P* = 0.475).

**Table 5:** Multivariable analysis of PNETs—clinicopathologic factors and survival

Variable	Hazard ratio (HR)	95 % CI for HR	*P* value
Tumor size >3 cm	0.475	0.143–1.580	0.475
Positive resection margins	5.234	1.030–26.595	0.046
High-grade tumor (G3)	4.276	1.099–16.638	0.036
WDCa	13.050	2.432–70.041	0.002
PDCa	9.237	1.505–56.679	0.016
TMN stage 3	16.173	1.258–207.994	0.032
TMN stage 4	18.127	1.628–201.869	0.018
Readmission	11.860	1.522–92.398	0.019

### Case Costing

Complete data from 123 PNET patients—56 who underwent LDP and 67 who underwent ODP— were available for analysis. The breakdown of the operative and hospital costs are described in Table 6. The LDP and ODP cohorts did not differ significantly in terms of median OR minutes (352 vs. 409 min, LDP vs. ODP, *P* = 0.065). Although the median total direct costs for the LDP group were 20 % lower, compared to the ODP group, this was not statistically significant (*P* = 0.179). Similarly, the median OR costs for the LDP group were 10.5 % higher than the median OR costs for the ODP group, but still not statistically significant (*P* = 0.091). Detailed analysis of OR costs revealed no statistically significant differences in terms of recovery room costs between the two groups (*P* = 0.466), but median OR team and median supply costs were respectively 20 % lower (*P* = 0.027) and 91.7 % higher (*P* < 0.001) for the LDP group. Median blood, nursing and laboratory costs were, respectively, 24.4, 25.6, and 38.4 % lower in the LDP group, and despite the statistical significance, this did not generate differences in the total direct costs between the groups. The remaining hospital costs, including costs for pharmaceuticals (*P* = 0.154), imaging (*P* = 0.526), and other costs (*P* = 0.317), were not significantly different between the two groups (Table 6).

**Table 6:** Breakdown of ratio and percentage change in median costs between LDP and ODP for PNETs

Variable	Ratio of median cost (LDP/ODP)	% change in median cost (LDP/ODP)	*P* value
Operating room minutes	0.84	−13.5 %	0.065
Total direct cost	0.80	−20.0 %	0.179
Operating room (OR) cost:	1.12	10.5 %	0.091
OR team	0.81	−20.0 %	0.027
Supplies	1.93	91.7 %	<0.001
Recovery room	0.83	−17.2 %	0.466
Blood cost	0.75	−24.4 %	0.020
Nursing cost	0.75	−25.6 %	0.048
Pharmacy cost	0.85	−15.3 %	0.154
Laboratory cost	0.61	−38.4 %	0.002
Radiology cost	0.52	−46.2 %	0.526
Other cost	0.97	−0.65 %	0.317

## Discussion

Many recent studies have shown that laparoscopic pancreatic surgery in well-selected groups of patients, as opposed to the open technique, is safe and associated with various equivalent or better surgical outcomes.^5^^–^ 9 ^,^ 23 ^–^ 26 However, there has been a steadily growing debate about the small size of these studies, the presence of significant selection biases, the lack of generalizability of results and the short-term follow-ups.^10^^–^ 14 In this study, we aim to put the controversy to rest on the impact of LDP for PNETs on postoperative, oncologic, and cost-analysis outcomes.

Few large series have compared surgical outcomes of LDP vs. ODP for PNET patients.^4^^,^ 5 ^,^ 18 The vast majority of the PNETs in our study were nonfunctioning (78 %), which is consistent with other reports in the literature.^18^ Of the 37 patients with functioning PNETs, a significantly higher percentage underwent ODPs. In accordance with their functioning tumors, patients undergoing ODP were more often symptomatic, with abdominal pain and hypoglycemia being the most common presenting symptoms. Patients with smaller neoplasms were more frequently chosen for LDP, which is commonly observed in previous similar reports.^5^^,^ 12

With regard to intraoperative outcomes, many series have shown that the duration of LDP is significantly longer when compared to ODP.^6^^,^ 15 ^,^ 27 ^–^ 29 However, our results show no significant differences between the duration of the two approaches. Discrepancies regarding surgical case duration may be attributed to differences in tumor size as well as differences regarding the various stages of disease, which may require a more or less extensive resection. Despite not having statistically significant differences between the LDP and ODP groups in terms of spleen preservation, our splenectomy rates were lower in the LDP group. Lower splenectomy rates after LDP reached a statistical significance in recent similar comparative series.^10^^,^ 14 This might be explained by a planned splenectomy due to higher rates of malignancy in the ODP group; in which case, proximity of tumor to the splenic vasculature often makes preservation difficult. Based on the literature, conversion rates of LDP may vary significantly. In this study, conversion rates were significantly lower compared to other series.^14^^,^ 30 This might be explained by our low rate of intraoperative complications in addition to the relative ease of identifying PNETs without needing intraoperative ultrasound. However, high rates of conversion highlight the demand for specific training in LDP.

Recent studies show no significant differences in terms of postoperative morbidity between the two techniques.^5^^,^ 11 ^,^ 13 ^–^ 15 ^,^ 28 However, some of these studies are not specific and include both benign and malignant pancreatic lesions. In contrast, our data support that PNET patients undergoing LDP have significantly lower rates of overall postoperative complications, including POPF, incisional hernia, intra-abdominal collection or abscess, bowel obstruction, and superficial or deep wound infection. Without reaching statistical significance, we observed that the LDP group experienced fewer POPFs compared to the ODP group, which is conflicting with recent results obtained by other investigators.^10^^,^ 11 ^,^ 13 ^,^ 14 However, some of these reports are underpowered, and others are large meta-analyses, which include studies of suboptimal quality. Moreover, in these studies, there is significant inconsistency regarding the definition of POPF and differences between the clinical grading of POPFs were not always assessed. Therefore, the real advantage of LDP on the rates of POPF remained an open issue. Our LDP group was also associated with less intraoperative blood loss and had significantly shorter hospital stays when compared to the ODP group. This short hospital stay favored accelerated recovery and simultaneously contributed to lowering the costs of hospitalization. Our analysis failed to demonstrate any significant differences between the two groups with regard to postoperative mortality, recurrence, reoperation, or readmission rates. Other comparative cohorts have recently reported no significant differences in terms of intraoperative blood loss and LHS,^6^^–^ 9 as well as similar rates of readmission and 30-day mortality.^5^^,^ 12

It has been frequently suggested that LDP has similar or even superior oncologic outcomes compared to the open technique, such as rates of R0 resections and survival; however, most of these studies are relatively small cohorts (<30 patients), they are often composed of nonhomogeneous samples in terms of patient and tumor characteristics, and assess only 2- or 3-year survival rates.^5^^,^ 6 ^,^ 10 ^,^ 11 ^,^ 13 ^,^ 16 ^,^ 28 ^,^ 29 A recent study shows that LDP for pancreatic adenocarcinoma does not compromise perioperative oncologic outcomes, but disease-specific and overall survival data are lacking.^12^ In contrast, our study is composed of 171 patients, all of whom were diagnosed with PNETs and 5-year survival rates are assessed. In analyzing oncologic outcomes between the two techniques, it is also crucial to note that we had no statistically significant differences in pathology between patients undergoing LDP vs. ODP. In contrast, in many similar series, the pathological characteristics between the two groups were significantly different. For example, the minimally invasive group had significantly smaller, lower grade tumors with less lymphovascular invasion, fewer regional nodal metastases, more well-differentiated tumors and lower stages of disease.^5^^,^ 12 ^,^ 16 Such relevant differences in pathology constitute the main reasons why recent comparative studies debate existing findings on the oncologic equivalency of the two techniques.^6^^,^ 31 ^–^ 33

The overall survival rate of the PNET patients in our cohort (90 %) is similar to other reports, where survival ranges between 65 and 90 % and was not significantly different between the LDP and ODP groups.^27^^,^ 34 Five-year survival rates were similar using the WHO and ENETS-TNM classification systems and was not influenced by type of resection—LDP vs. ODP. Nine patients with a WDCa and four patients with a PDCa underwent R0 LDP without postoperative morbidity or mortality at 5 years. Only one of these patients developed a tumor recurrence at 21 months from surgery and was alive at the conclusion of study follow-up, at 8 years from surgery. Overall, three patients in the LDP group (4 %) developed a recurrence after R0 resection. Additionally, three patients in the LDP group—two of whom had WDTs and one with PDCa—developed liver metastases, which were treated with chemotherapy, and two of these patients were alive at the conclusion of study follow-up.

Along with the classification and staging of PNETs, we have examined pathologic characteristics that predicted poor survival. Using univariate analysis, we found that tumors >3 cm in diameter, high-grade tumors (G3), as well as positive resection margins predicted poor survival. Consistent with other data published,^27^^,^ 35 we found that WDCa, PDCa, and TNM stages 3 and 4 were also statistically associated with poor survival. On multivariable analysis, when controlling for demographics and clinicopathologic characteristics, tumor size >3 cm in diameter no longer correlated with poor survival; however, the remaining statistically significant factors on univariate analysis all maintained their significant association with poor survival. Interestingly, we found that 30-day readmission was a predictor of poor survival on multivariable analysis.

Although questions have been raised concerning the cost of the laparoscopic approach, large studies comparing direct costs after LDP vs. ODP are scarce. Therefore, the economic advantages of laparoscopic pancreatectomy are not settled. Comparative small-sized trials report similar total direct costs between LDP and ODP,^8^^,^ 36 but in contrast, a recent study shows that the laparoscopic approach is associated with extra cost.^15^ We demonstrated that LDP in comparison to ODP is cost-neutral. Although the total direct costs, including nursing, pharmacy, laboratory, and radiology costs were lower in the LDP than the ODP group, these were not statistically significant. Due to much higher supply expenses, median OR costs were higher for the laparoscopic group than the open approach, but still not significantly so.

As with any retrospective review, this study has several limitations. Although there were no statistically significant differences in pathologic characteristics between the two groups, smaller- and lower-grade PNETs without locoregional or distant metastases were selected for the laparoscopic approach. As a result, there are relatively more malignant tumors in the ODP group, though this is consistent with other reports.^5^^,^ 25 We additionally acknowledge the relative difficulty of comparing data in patients undergoing distal pancreatectomy, given that multiple factors may play into the decision to use the open or the laparoscopic approach. Lastly, our study population is defined by the demographics seen at our institution, a high-volume academic center with experienced pancreatic surgeons, and therefore results may be less applicable at other institutions.

## Conclusion

We conducted this study to address the current significant controversy over the surgical outcomes of LDP vs. ODP as treatment for pancreatic cancer. Based on our research, LDP for PNETs presents similar long-term oncologic outcomes as compared to ODP, providing reduced overall morbidity in terms of postoperative complications without compromising survival. Advanced WHO classification and TNM stage, as well as positive resection margins, advanced tumor grade, and 30-day readmission are predictors of poor survival for PNET patients. Additionally, LDP is cost-neutral as opposed to the open approach and yields shorter hospital stays, less postoperative pain, better cosmetic results, and faster postoperative recovery. Larger prospective controlled trials are needed to further validate the advantages of LDP in well-selected groups of patients.
